# Contemporary management of brain arteriovenous malformations in mainland China: a web-based nationwide questionnaire survey

**DOI:** 10.1186/s41016-020-00206-0

**Published:** 2020-09-01

**Authors:** Yu Chen, Xiangyu Meng, Li Ma, Yang Zhao, Ye Gu, Hengwei Jin, Dezhi Gao, Youxiang Li, Shibin Sun, Ali Liu, Yuanli Zhao, Xiaolin Chen, Shuo Wang

**Affiliations:** 1 Department of Neurosurgery, Beijing Tiantan Hospital, Capital Medical University, Beijing, 100070 China; 2 Department of Interventional Neuroradiology, Beijing Tiantan Hospital, Capital Medical University, Beijing, China; 3 Department of Neurosurgery, Peking University International Hospital, Peking University, Beijing, China; 4 Department of Gamma-Knife Center, Beijing Tiantan Hospital, Capital Medical University, Beijing, China

**Keywords:** Arteriovenous malformation, Questionnaire survey, Mainland China

## Abstract

**Background:**

In the benefit of the large population and rapid economic growth, the interventional techniques and equipment for brain arteriovenous malformations (bAVMs) in mainland China have been rapidly improved. Chinese neurosurgical cerebrovascular physicians have accumulated rich experience and made pioneering explorations. This study aims to summarize the experience and treatment progress of bAVMs in mainland China.

**Methods:**

We performed a web-based nationwide questionnaire survey among 67 tertiary neurosurgical institutions that had acknowledged treating bAVMs in the primary survey. Our questionnaire included clinical characteristics, radiological findings, intervention indications/contraindications, intervention timing, and intraoperative management of different treatment modalities.

**Results:**

A total of 63 participants from 49 (73.1%) tertiary neurosurgical institutions responded to our questionnaire. Forty-two (66.7%) were neurosurgeons, 13 (20.6%) were neurointerventionists, and 8 (12.7%) were radiosurgeons. Approximately 3500 to 4000 cases of bAVMs were treated annually in these 49 departments. All participants agreed that the conclusions of ARUBA are debatable. Flow-related aneurysms, deep venous drainage, and arteriovenous fistula were considered as common hemorrhagic risk factors. Unruptured SM IV-V bAVMs, giant bAVMs, pediatric bAVMs, elderly bAVMs, and eloquent bAVMs were not absolute contraindications to intervention. Maximum lesion occlusion and minimal functional impairment were the principles of intervention management. Most of the neurosurgeons and neurointerventionists recommended early intervention (< 30 days) for ruptured bAVMs, and the radiosurgeons suggested intervention in the chronic phase or recovery phase (*P* < 0.01) and preferably 3 months after bleeding. Multi-modality strategies were thought effective for complex bAVMs, and more exploration of individualized intraoperative management was necessary.

**Conclusions:**

Intervention was acceptable for specific selected unruptured bAVMs in mainland China, especially in patients with hemorrhagic risk factors. The application of multidisciplinary cerebrovascular team and multicenter large-sample international registry study might be the next work for Chinese neurosurgical cerebrovascular physicians.

## Background

Brain arteriovenous malformations (bAVMs) are complex and rare cerebral vascular dysplasia. The main purpose of treatment is to avoid the neurological impairment caused by hemorrhagic stroke [1]. In general, ruptured bAVMs require aggressive intervention, but the treatment strategy for unruptured bAVMs was still controversial. In 2014, a randomized trial (ARUBA) indicated that medical management alone is superior to interventional therapy for the prevention of death or stroke in unruptured bAVMs [2]. However, the conclusion was opposite with many published studies [3, 4]. Nowadays, most cerebrovascular neurosurgical centers around the world did not change the treatment indications and intervention strategies of unruptured bAVMs due to the results of ARUBA. With the development of new intervention strategies, bAVMs may have more opportunities to achieve more aggressive strategies. In the benefit of the large populations and rapid economic growth, the interventional techniques and equipment in mainland China have been rapidly improved. Chinese neurosurgical cerebrovascular physicians have accumulated rich experience and made pioneering explorations. In this study, we conduct a nationwide questionnaire survey to summarize the experiences and treatment progress of bAVMs in mainland China.

## Methods

### Study design and assessed items

Before the questionnaire survey, we conducted a preliminary survey (by the recommendation of the Chinese Medical Association Neurosurgery Branch and the suggestion of the corresponding author Dr. Chen and Dr. Wang) to screen out the largest bAVM diagnosis treatment center in the local provinces. And finally, a web-based questionnaire (Additional file 1) surveying details of bAVMs was sent to 67 nationwide tertiary neurosurgical institutions via email and WeChat QR code. We used logic operation which skipped certain questions based on specific answers. Thus, we only let participants answer questions in their field (microsurgery, embolization, radiosurgery). Radiosurgeons were affiliated with the Department of Radiosurgery. In this study, the radiosurgeon was engaged in Gamma knife radiosurgery for bAVMs. The first few questions were focused on general characteristics of participants, such as department, hospital, years of experience, and number of treated bAVMs per year. We classified the questions concerned by all 3 departments as general questions, such as clinical incidence, radiological findings, intervention indications/contraindications, and intervention timing. More detailed questions in subgroups were followed, including intraoperative details and perioperative management. All variables relating to bAVMs (including the definition of clinical and morphological baseline characteristics) are defined according to currently recommended reporting terminology for clinical bAVMs research [5]. The study was performed according to the national law, institutional ethical standards, and guidelines of the Helsinki Declaration.

### Statistical assessment

The categorical variables are presented as counts (with percentages). The Pearson chi-square test or Fisher exact test was used to compare categorical variables as appropriate. Two-tailed *t* tests were employed to compare continuous variables (normal distribution variable). Wilcoxon rank-sum test was applied to compare non-normal distribution continuous variables. *P* value < 0.05 was considered to be statistically significant. Statistical analysis was performed using SPSS (version 25.0, IBM, New York, USA).

## Results

**Fig. 1:**
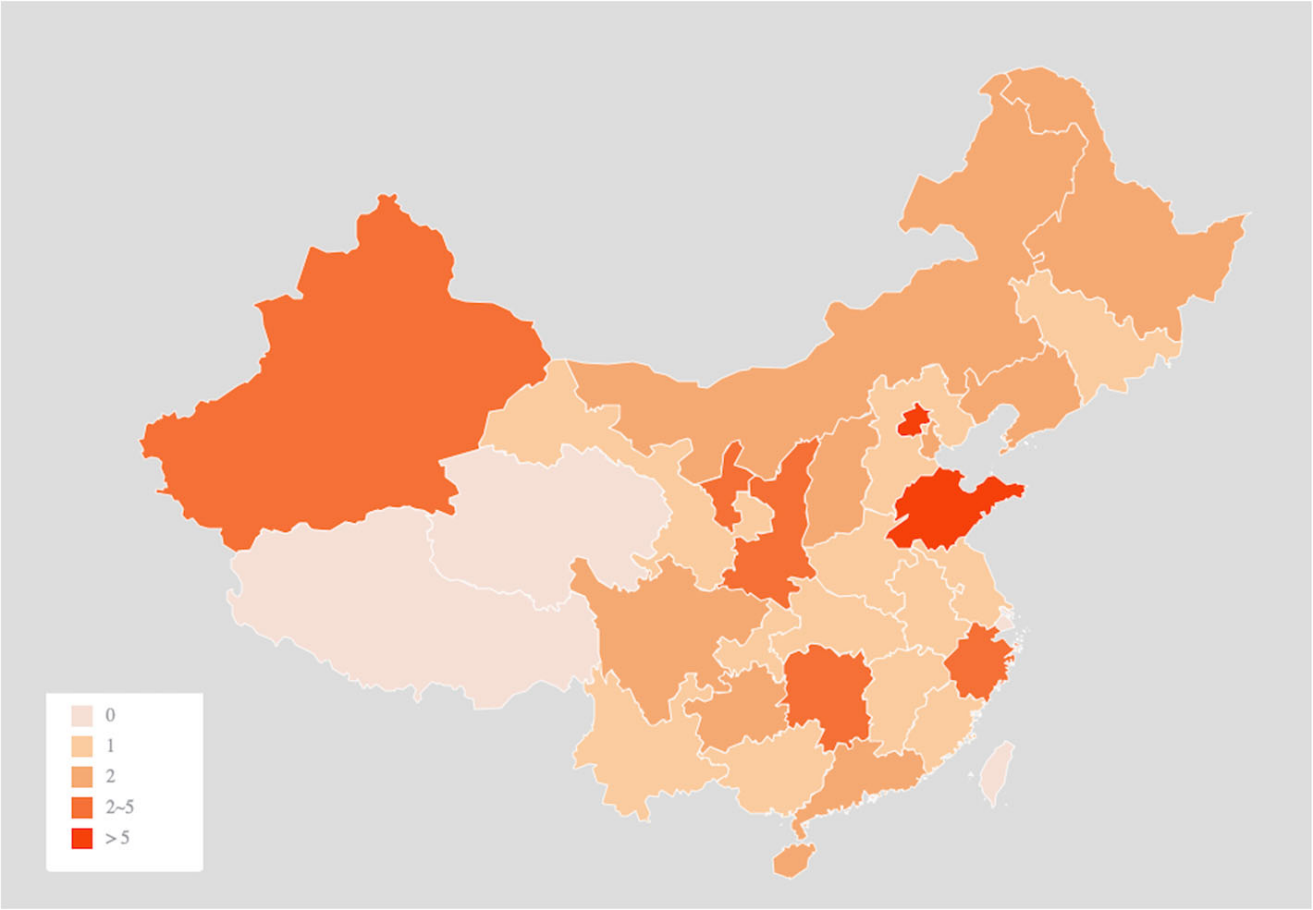
The distribution of participating departments throughout the country

A total of 63 participants from 49 (73.1%) institutions responded to our questionnaire (Fig. 1). Thirty-eight participants (60.3%) were department directors, and 62 participants (98.4%) have more than 5 years of experience in the clinical treatment of bAVMs. Of the 63 participants, 42 (66.7%) were neurosurgeons, 13 (20.6%) were neurointerventionists, and 8 (12.7%) were radiosurgeons. Approximately 3500 to 4000 cases of bAVMs were treated annually in these 49 departments.

### General Questions

#### Microsurgery, embolization, radiosurgery (63 participants)

1. Did you agree with the conclusions of ARUBA that conservative management is better than intervention for unruptured bAVMs, and could these conclusions be generalized to all unruptured bAVMs (single-choice question)?

All participants (100.0%) agree that the conclusions of ARUBA are debatable and cannot be generalized to all unruptured bAVMs.

2. What do you think are the risk factors for subsequent hemorrhage in unruptured bAVMs (multichoice question)?

**Fig. 2:**
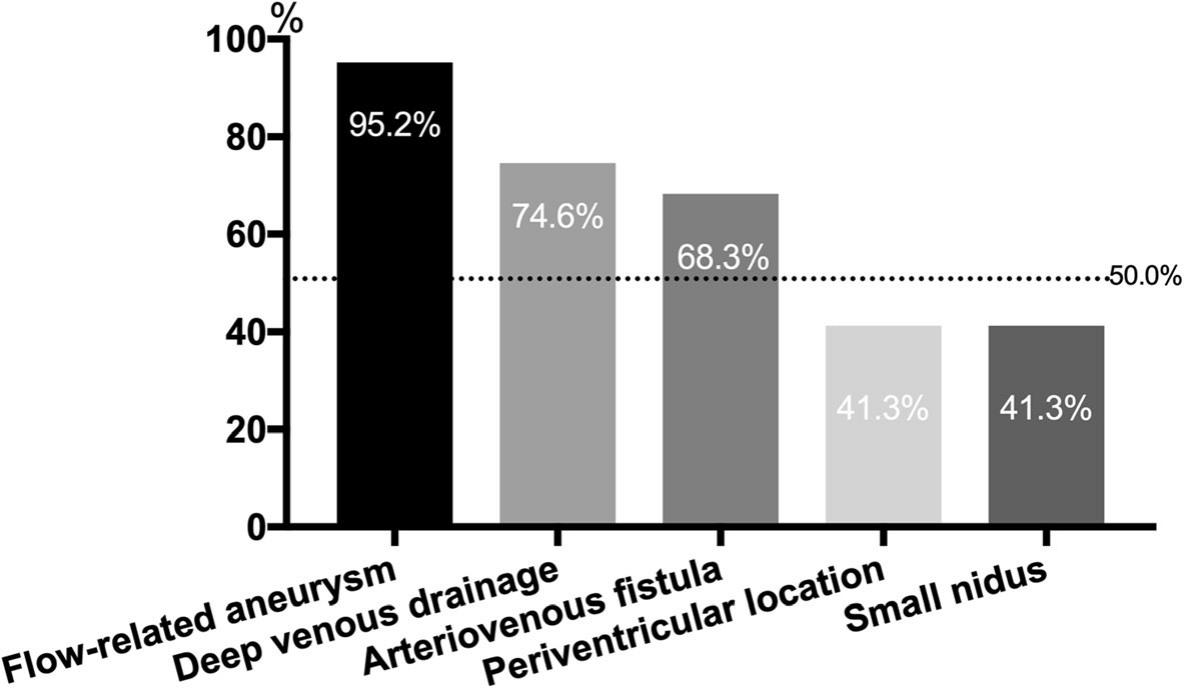
Hemorrhagic risk factors in unruptured bAVMs

Flow-related aneurysms (60 participants, 95.2%), deep venous drainage (47 participants, 74.6%), arteriovenous fistula (AVF) (43 participants, 68.3%), periventricular location (26 participants, 41.3%), and small nidus (26 participants, 41.3%) were considered as risk factors for subsequent hemorrhage of unruptured bAVMs (Fig. 2). There was no difference of opinion among the three departments (*P* > 0.05).

3. Do you think unruptured Spetzler-Martin (SM) grades IV–V bAVMs are the interventional contraindications (single-choice question)? If not, which kind of unruptured SM IV-V bAVMs should take intervention management (multichoice and open question)?

**Fig. 3:**
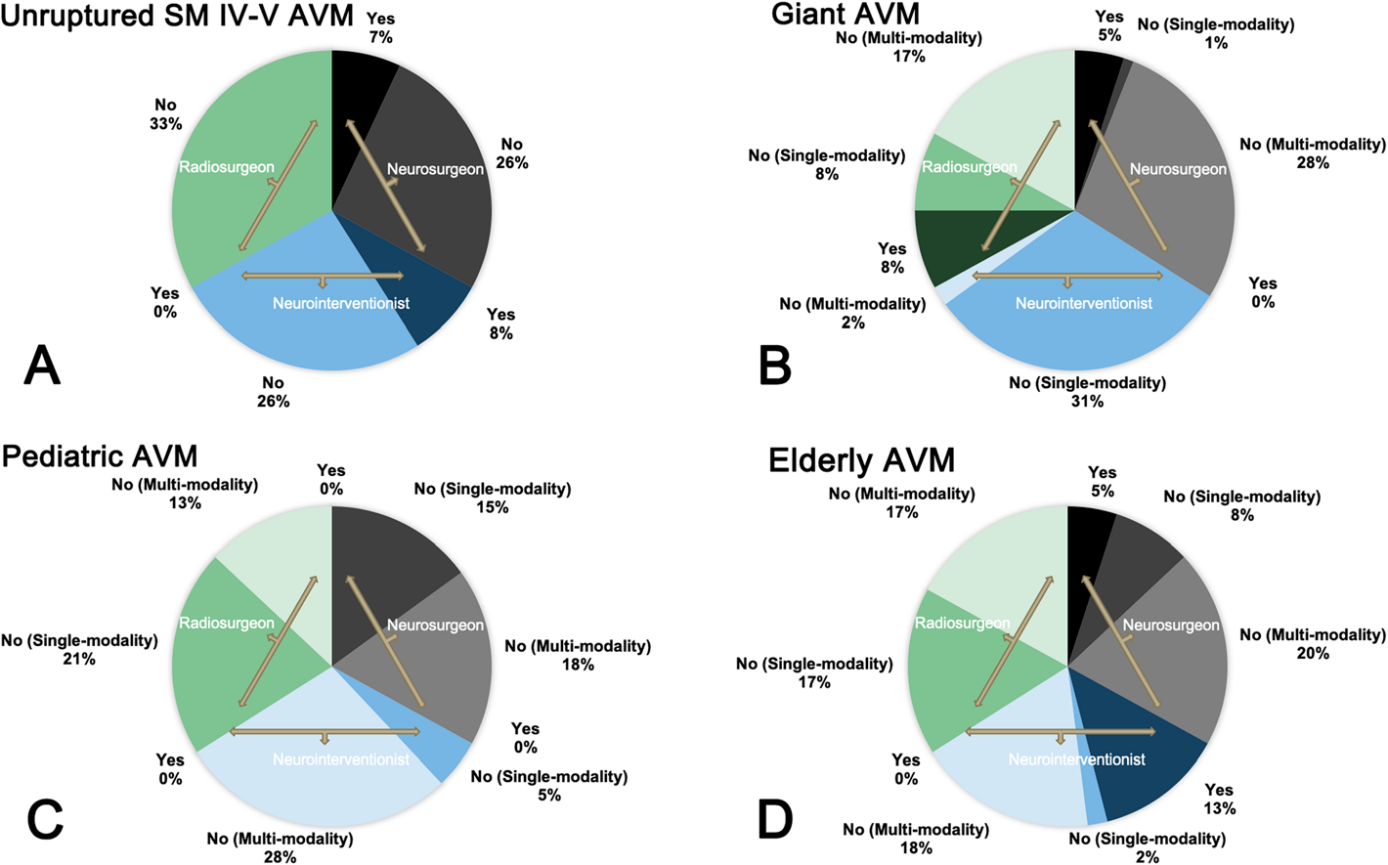
Interventional indications and contraindications of unruptured SM IV–V bAVMs, giant bAVMs, pediatric bAVMs, and elderly bAVMs in different departments

The majority of participants (44 participants, 69.8%) suggested intervention for specific SM grades IV–V unruptured bAVMs, especially in patients with high hemorrhagic predictors (Fig. 3a). There was no difference of opinion among the three departments (*P* = 0.942). Besides, younger bAVMs (41 participants, 65.1%) were also recommended positive intervention because of the high cumulative subsequent rupture risk.

4. Do you think giant bAVMs (> 6 cm) are the contraindication of intervention (single-choice question)? If not, which kind of intervention modality do you recommend (multichoice question)?

Fifty-five participants (87.3%) thought giant bAVMs were not interventional contraindications, and multi-modality intervention strategy was recommended by 40 participants (72.7%) (Fig. 3b). But the multi-modality combinations were different within three departments, combined embolization and microsurgery (61.1%) was the most preferred strategy of the neurosurgeons, and combined embolization and radiosurgery (66.7%) was more preferred by the radiosurgeons. However, target embolization (58.3%), including embolization of cerebral aneurysms and high-flow AVFs, was the most preferred intervention strategy for giant bAVMs among neurointerventionists.

5. Do you think pediatric bAVMs are the contraindication of intervention (single-choice question)? Do you think pediatric intervention strategies should be more aggressive than adults (single-choice question)? And which kind of intervention modality do you recommend for pediatric bAVMs (multichoice question)? Do you agree that the purpose of intervention in pediatric bAVMs is complete nidus obliteration and maximum functional protection (single-choice question)?

No participants (0.0%) agreed pediatric bAVMs were interventional contraindication. Forty-six participants (73.0%) thought the pediatric bAVMs should undergo more aggressive management than adults, and 37 participants (58.7%) recommended multi-modality intervention strategies for pediatric bAVMs (Fig. 3c). There was no difference of opinion among the three departments (*P* = 0.055). Fifty-seven participants (90.5%) agree that the purpose of intervention in pediatric bAVMs is complete nidus obliteration and maximum functional protection.

6. Do you think elderly bAVMs (> 65 years) are the contraindication of intervention (single-choice question)? If no, which kind of intervention modality do you recommend (multichoice question)? Do you recognize that partial occlusion and target embolization concentrated on hemorrhagic risk factors were more recommended than complete obliteration for elderly bAVMs (single-choice question)?

Fifty-one participants (81.0%) thought elderly bAVMs still need interventions. However, unlike adult patients, multi-modality strategies (36 participants, 70.6%) such as combined embolization and microsurgery (10 participants, 19.6%) or combined embolization and radiosurgery (26 participants, 51.0%) were more preferred than single modalities (15 participants, 29.4%) (*P* < 0.01, Fig. 3d). Besides, unlike pediatric patients, partial occlusion and target embolization (23 participants, 45.1%) concentrated on hemorrhagic risk factors were also recommended, rather than complete obliteration.

7. Do you think eloquent AVMs are the contraindication of intervention (single-choice question)? Which intervention modality do you prefer (multichoice question)?

**Fig. 4:**
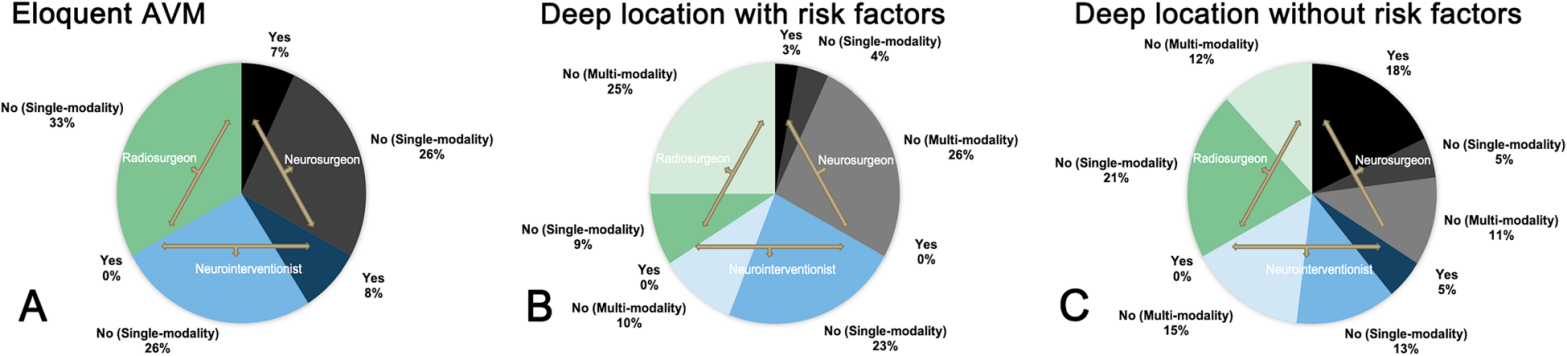
Interventional indications and contraindications of eloquent bAVMs in different departments

Most of the participants (51 participants, 81.0%) agreed that eloquent AVMs are decisive in their decision on treatment, but the interventional indications need to be personalized. Thirty-three neurosurgeons (78.6%) proposed that only specific selected eloquent bAVMs would be recommended for microsurgical resection. Ten neurointerventionists (76.9%) consider that target embolization on the hemorrhagic risk factors was more preferred for bAVMs located in the eloquence area. All radiosurgeons (100.0%) believe that the Gamma knife has unique advantages for eloquent bAVMs, especially in small to moderate-sized and compact nidus (Fig. 4a).

8. What treatment strategy would you prefer for bAVMs with/without hemorrhagic risk factors located in the internal capsule, basal ganglia, thalamus, and brainstem (multichoice and open question)?

For bAVMs with hemorrhagic risk factors, most of the participants (59 participants, 93.7%) agreed with intervention treatment, and combined embolization and radiosurgery (65.1%) was the first-line recommended strategy. Besides, target embolization was suggested by 16 participants (25.4%) (Fig. 4b). Fourteen neurosurgeons (33.3%) consider surgical resection as an alternative, especially for bAVMs with emergency hemorrhage.

For bAVMs without hemorrhagic risk factors, about half of the neurosurgeons (22 participants, 52.4%) preferred conservative management, but the majority of neurointerventionists (84.6%) and radiosurgeons (100.0%) suggested intervention treatment. Among the 39 participants who supported the intervention, 27 participants (69.2%) recommended radiosurgery (41.0%) or combined embolization and radiosurgery (28.2%) (Fig. 4c).

9. When do you think is the best intervention timing in patients with stable ruptured bAVMs (vital signs are stable, no obvious signs of cerebral hernia) (single-choice question)? Acute phase (within 48 h)/subacute phase (2 days to 1 month)/chronic phase (1 month to 3 months)/recovery phase (> 3 months)

**Fig. 5:**
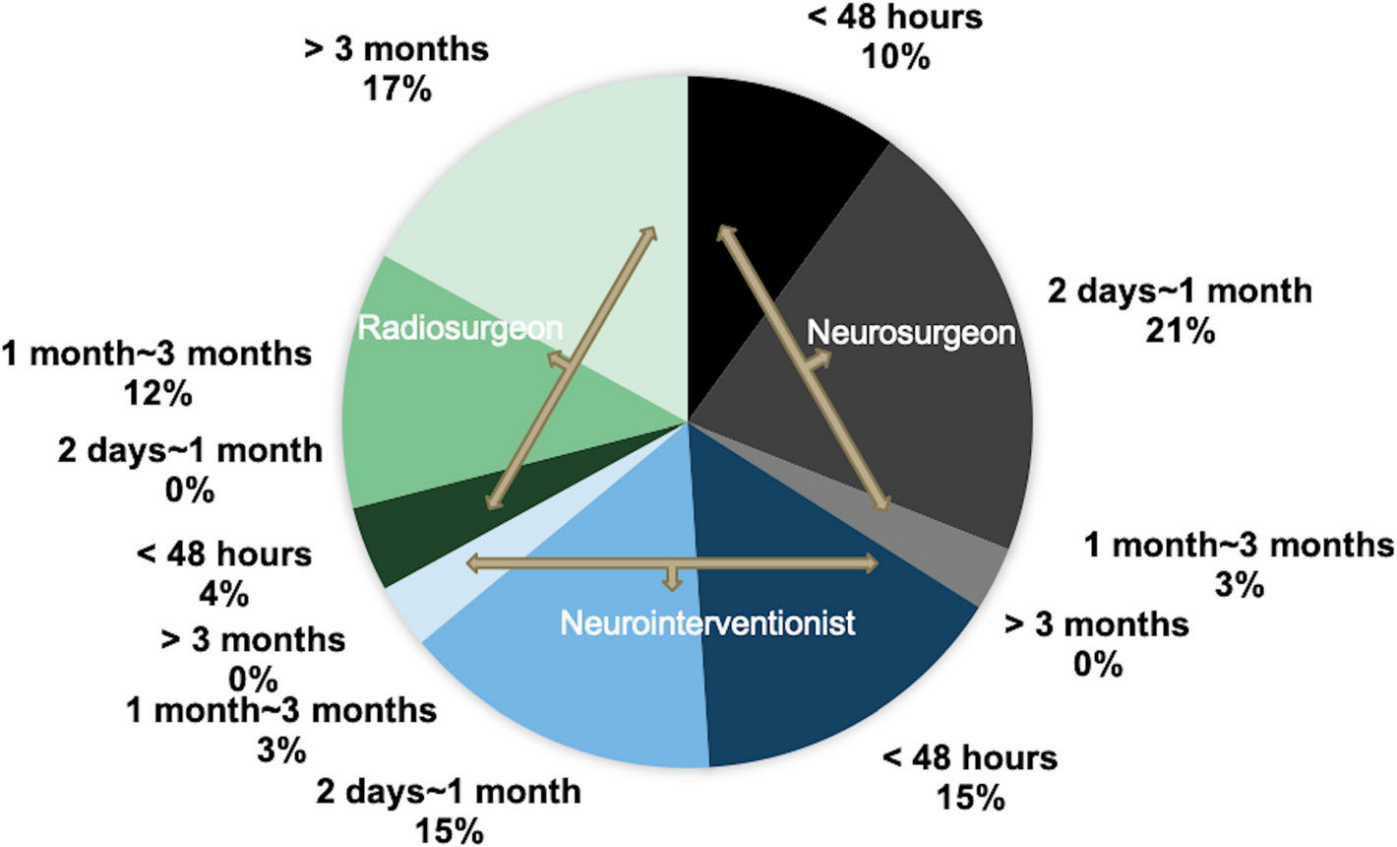
Intervention timing recommended by different departments

There were significant differences in the timing of intervention among the three departments (*P* < 0.01). Most neurosurgeons (90.5%) and neurointerventionists (92.3%) did not recommend intervention in the chronic phase or recovery phase. In the subgroup of neurosurgeons, 12 participants (28.6%) recommended surgery during the acute phase, and 26 participants (61.9%) preferred the subacute phase. However, considered the high subsequent hemorrhagic risk and the advantages of target embolization on hemorrhagic risk factors, 6 of neurointerventionists (46.2%) suggested embolization during the acute phase. Unlike either, the majority of radiosurgeons (87.5%) suggested intervention in the chronic phase or recovery phase and preferably 3 months after bleeding (Fig. 5).

### Subgroup detail questions

#### Microsurgery, embolization (55 participants)

10. Do you think that the single-stage combined embolization and microsurgery strategy is beneficial (single-choice question)? If yes, which of the following is the most important (single-choice question)? Intraoperative angiography (clarify angioarchitecture characteristics and avoid lesion residue)/intraoperative embolization. If you select intraoperative embolization, which embolization strategy do you prefer (multichoice and open question)?

All 55 neurosurgeons and neurointerventionist (100.0%) agreed that single-stage combined embolization and microsurgery is beneficial. Most of them (60.0%; 24 neurosurgeons, 57.1%; 9 neurointerventionists, 69.2%) considered intraoperative embolization as the most significant advantage because of the reduced flow and volume of the nidus. Besides, intraoperative target embolization for the hemorrhagic predictors (69.1%) and the deep part of the nidus (58.2%) was the most recommended intraoperative embolization strategies. Only 29.1% of the participants suggested excessive embolization.

11. Which of the following areas do you prefer to embolize in the single-stage combined embolization and microsurgery (single-choice question)? The feeding arteries/the feeding arteries and nidus. And what is the reason of your choice (open question)?

Different departments have different views on this question. Twenty-eight neurosurgeons (66.7%) believed that embolization of the feeding artery was the most advantageous intraoperative embolization strategy because of the reduced blood supply, and the embolization of the nidus should be avoided, especially in deep lesions, because the lesions after embolization would become hard and difficult to be exposed intraoperatively. However, most of the neurointerventionists (8 of 13 participants, 61.5%) believed embolization of the feeding artery and nidus is more beneficial because it can reduce the nidus volume and reduce the intraoperative blood loss.

#### Embolization (13 participants)

12. What is the embolic material routinely used for bAVMs in your department (single-choice question)? And which embolization strategy do you think is more beneficial (single-choice question)? Embolize hemorrhagic risk factors/embolize hemorrhagic risk factors and the nidus

All participants (100.0%) preferred Onyx (eV3, Inc.) as their first choice when embolizing bAVMs. Most of the participants (12 participants, 92.3%) preferred to embolize hemorrhagic risk factors and nidus.

#### Radiosurgery (8 participants)

13. What is the minimum margin dose you suggested for single-stage radiosurgery (open question)?

Five participants (62.5%) suggested 16 Gy as the minimum margin dose for single-stage radiosurgery, while 3 participants (37.5%) suggested 18 Gy.

14. Do you agree that pre-radiosurgery embolization is not conducive to the subsequent obliteration after radiosurgery (single-choice question)? Which kind of bAVMs do you think would benefit from pre-radiosurgery embolization (multichoice and open question)?

All participants (100.0%) consider the pre-radiosurgery embolization might reduce the obliteration rate. However, they still recommended pre-radiosurgery embolization for specific selected bAVMs, such as patients with hemorrhagic risk factors (87.5%), high flow (37.5%), and large-volume nidus (62.5%).

## Discussion

Brain arteriovenous malformations are rare vascular lesions with the presentation of hemorrhagic stroke, seizure, headache, and focal neurological deficit [6]. Despite the incidence was scarcity, bAVMs account for the majority of spontaneous intracranial hemorrhage in children and young adults [7]. The SM grading system is widely used to estimate the risk of postoperative complications [8]. Generally, for ruptured bAVMs, intervention was recommended regardless of grade. However, the management of unruptured bAVMs was still controversial. To summarize the current status and experiences of the treatment of bAVMs in mainland China, we conducted a web-based nationwide questionnaire survey among 49 tertiary neurosurgical institutions (63 participants) including microsurgery, embolization, and radiosurgery.

Although ARUBA indicated negative outcomes of intervention than conservative management in unruptured bAVMs [2], all participants in this study still support intervention for specific selected bAVMs. The main criticisms of the ARUBA trial included insufficient follow-up period (33 months), heterogeneity and uneven distribution between treatment modalities, low obliteration rate, small sample size, and unusual high incidence of post-interventional complication [9]. These deficiencies make ARUBA’s findings cannot be recognized as the first-line evidence to select conservative management or intervention of all unruptured bAVMs. Previous studies proposed that elevations of vascular endothelial growth factor (VEGF) or alterations in the vascular wall [10] and many hemodynamic changes, such as flow-related aneurysms [11], abnormally high blood flow through shunting [12], smaller bAVMs, a single and/or stenosed draining vein, and so on [13], may contribute to bAVMs rupture. In this study, the hemorrhagic hemodynamic factors were similar to previous studies, which means that most of the participants in mainland China share the same view on the hemorrhagic risk factors.

### Interventional indications and contraindications

Two recent completed randomized clinical trials or prospective registries (ARUBA and SAIVM) suggested that the risk of intervention may outweigh the risk of future rupture for unruptured bAVMs [2, 14]. Therefore, the selection of interventional indications has become more cautious clinically, especially in unruptured patients. Generally, SM grades IV–V bAVMs are recommended to observed unless ruptured [15]. However, previous studies calculated an average rate of 15% for persisting disability and a risk of 15% death after bAVMs hemorrhage [16]. The outcomes of the hemorrhage were a permanent downgrade in function to an mRS score > 1 in 88% and an mRS score > 2 in 69% [3]. Therefore, for patients with hemorrhagic risk factors and younger age, more aggressive treatment might be recommended because of the high cumulative subsequent rupture risk [3, 16].

The ARUBA trial did not include giant bAVMs, and it is still controversial whether the intervention can benefit giant bAVMs in other previous studies [2]. Yang et al. indicated that interventions for giant bAVMs should be considered cautiously because the hemorrhagic risk is similar and functional outcomes may be better in the conservatively managed population [17]. In contrast, Chang et al. proposed that selected symptomatic patients with giant bAVMs can be treated successfully with good outcomes and acceptable risk [18]. Recently, Reinard et al. found that good outcomes are attainable with a multimodal treatment approach in carefully selected patients with giant bAVMs [19]. Besides, further study indicated that radiosurgery after microsurgery or embolization might be the most advantageous strategy [20]. In this study, most participants considered that intervention for giant bAVMs was desirable, and multi-modality strategy was more preferred, especially in patients with hemorrhagic risk factors.

Only adult bAVMs were included in the ARUBA trial [2]. However, due to the long life expectancy, the high cumulative lifelong hemorrhagic risk and better neural plasticity of pediatric bAVMs, the ARUBA conclusion that medical management alone has a better prognosis may not apply to pediatric bAVMs. In fact, pediatric bAVMs were often treated more aggressively than adults [21]. Minimum trauma and maximum occlusion were the intervention principle for pediatric bAVMs [22]. Similar to giant bAVMs, multi-modality strategy was more preferred for pediatric bAVMs in this study. On the other hand, another special population corresponding to pediatric bAVMs is elderly bAVMs (> 65). Nowadays, whether elderly bAVMs should undergo intervention treatment is still controversial. Many previous studies proposed that elderly bAVMs were interventional contraindication, because only a life expectancy over 20 years could be a prerequisite for treatment [20], and the risk of treatment can outweigh the risk of bleeding. However, Pabaney et al. proposed surgical management of elderly bAVMs can result in complete obliteration and acceptable clinical outcomes, with an overall mortality rate of 3.6% and an obliteration rate of 87% [23]. In this study, most participants agreed that elderly patients should receive intervention management, but more minimally invasive and lower risk strategies were recommended, such as partial occlusion of hemorrhagic risk factors.

Eloquence has always been considered to be closely related to the postoperative neurological outcomes. No matter in the SM Grade Scale [8], or the Lawton-Young Grade Scale [24], or the new prediction scale of postoperative neurofunctional deficit-HDVL Grade Scale [25], eloquence is the key evaluation criteria. Generally, limited visual deficit and mild aphasia were acceptable because of the small impact on the quality of life [20], and bAVMs in the deep location or central motor cortex were considered as surgical contraindication [26, 27]. Lesion-to-eloquence distance (LED) < 4.95 mm was indicated as an independent predictor for postoperative neurofunctional deficits [25]. Target embolization for hemorrhagic risk factors was considered feasible in eloquent bAVMs by neurointerventists because of the less risk of postoperative neurofunctional deficits and effective reduction of subsequent hemorrhagic risk [28]. Besides, most previous studies suggested that radiosurgery has unique advantages for eloquent bAVMs, especially in small to moderate-sized and compact nidus [29].

Giant bAVMs, pediatric bAVMs, elderly bAVMs, and eloquent bAVMs are relatively rare in the overall bAVMs population. The current studies are all retrospective studies based on a small sample size, which cannot provide high-level management evidence for such patients. Further multi-center randomized controlled trials with larger sample size are needed for this particular bAVMs.

### Current single-modality and multi-modality strategies

Available treatment strategies for bAVMs currently include medical management, microsurgical resection, endovascular embolization, stereotactic radiosurgery, or combination thereof. However, there is still no consensus on the timing of intervention for ruptured bAVMs. Ahmad et al. found that the time interval between AVM bleeding and surgery did not influence early or late outcomes [30]. Martinez et al. recommended a delayed intervention for at least 4 weeks after the initial hemorrhage. In contrast, Deng et al. proposed that short-term outcomes of the early intervention were better, though the long-term outcomes were similar [31]. In this study, most of the neurosurgeons and neurointerventionists recommended early intervention (< 30 days) for ruptured bAVMs; however, the radiosurgeons suggested intervention in the chronic phase or recovery phase and preferably 3 months after bleeding. Therefore, we speculated that different intervention strategies may have different optimal intervention window periods for ruptured bAVMs.

Microsurgical resection is currently considered as the first-line strategy for low-level (SM grades I–II) and superficial lesions because of the highest rates of complete cure with an acceptable safety profile [32], especially in emergency patients [33]. However, previous studies reported that the risk of a serious neurologic deficit increases dramatically for SM grades III and IV/V bAVMs to 17% and 45% [3, 32], respectively. Curative embolization of bAVMs is difficult to achieved, and previous studies have suggested that > 25% embolization in a single session might be associated with an increased risk of perioperative complications [34]. Recently, target embolization of discrete hemorrhagic risk factors was indicated that embolization of nidal or perinidal aneurysms can reduce recurrent hemorrhage within the first year following initial hemorrhage [28, 35], and transvenous embolization of selected bAVMs could achieve high angiographic obliteration rate [36]. Radiosurgery causes denaturation of endothelial cells and proliferation of vascular smooth muscle by radiation, which blocks or compresses the vascular lumen [37]. But the increased hemorrhagic risk between treatment-occlusion interval and radiation-induced complications (RICs) may limit the application of radiosurgery [38].

Multi-modality strategies are often recommended for complex bAVMs. Generally, embolization is usually used as an adjunct therapy to reduce a bAVM’s volume before radiosurgery or reduce the nidus blood flow before resection at present [39, 40]. However, the increased hemodynamic stress in the remain lesions after invasive treatment for partial nidus may induce an increased hemorrhagic risk in the treatment interval [41], and pre-radiosurgery embolization has been demonstrated that may cause the lesions divided into distinct compartments, finally inducing a negative impact on obliteration [42]. Single-stage combined embolization and microsurgery in the hybrid angio-surgical operating room might an efficient strategy for complex bAVMs to reduce the complications in the treatment interval and detect the residual lesions in time [43, 44]. In this study, we found that for the same type of bAVMs, the optimal intervention strategy proposed by different subspecialty departments often differ. Therefore, further studies are required to organize multicenter international trials to explore the most optimal and individualized intervention strategies for bAVMs.

Several potential limitations of this study should be noted. Firstly, this is a web-based questionnaire survey that prevents all participants from having in-depth discussions face-to-face. Secondly, the uneven proportion of doctors in the three departments may lead to deviations in the conclusions. Thirdly, there may be inconsistent opinions due to different economic conditions and technological capabilities in each region. However, this study still reflects the current experience and diagnosis and treatment of bAVMs in most parts of mainland China.

## Conclusions

Intervention management was acceptable for specific selected unruptured bAVMs in mainland China, especially in patients with hemorrhagic risk factors. A multidisciplinary team for cerebrovascular diseases is necessary to develop optimal individualized strategies according to the clinical characteristics, angioarchitecture characteristics, and hemodynamic characteristics of patients.

## Supplementary information

**Additional file 1.** Contemporary web-based nationwide questionnaire survey on brain arteriovenous malformations management in mainland China

## Data Availability

All data relevant to the study are included in the article or uploaded as supplementary information.

## Abbreviations

bAVMs: Brain arteriovenous malformations
AVF: Arteriovenous fistula
SM: Spetzler-Martin
VEGF: Vascular endothelial growth factor
LED: Lesion-to-eloquence distance
HHT: Hemorrhagic telangiectasia
RICs: Radiation-induced complications

## References

[1] Klopfenstein JD, Spetzler RF (2005). Cerebral arteriovenous malformations: when is surgery indicated?. Acta Neurochir (Wien)..

[2] Mohr JP, Parides MK, Stapf C, Moquete E, Moy CS, Overbey JR (2014). Medical management with or without interventional therapy for unruptured brain arteriovenous malformations (ARUBA): a multicentre, non-blinded, randomised trial. Lancet..

[3] Bervini D, Morgan MK, Ritson EA, Heller G (2014). Surgery for unruptured arteriovenous malformations of the brain is better than conservative management for selected cases: a prospective cohort study. J Neurosurg..

[4] Ding D, Starke RM, Kano H, Lee JYK, Mathieu D, Pierce J (2017). Radiosurgery for unruptured brain arteriovenous malformations: an international multicenter retrospective cohort study. Neurosurgery..

[5] Joint Writing Group of the Technology Assessment Committee American Society of I, Therapeutic N, Joint Section on Cerebrovascular Neurosurgery a Section of the American Association of Neurological S, Congress of Neurological S, Section of S, the Section of Interventional Neurology of the American Academy of N, et al. (2001). Reporting terminology for brain arteriovenous malformation clinical and radiographic features for use in clinical trials. Stroke..

[6] Ding D, Liu KC (2014). Orbital venous congestion: rare manifestation of an intracranial arteriovenous malformation. J Clin Neurosci..

[7] Al-Shahi R, Fang JS, Lewis SC, Warlow CP (2002). Prevalence of adults with brain arteriovenous malformations: a community based study in Scotland using capture-recapture analysis. J Neurol Neurosurg Psychiatry..

[8] Spetzler RF, Martin NA (1986). A proposed grading system for arteriovenous malformations. J Neurosurg..

[9] Goldberg J, Raabe A, Bervini D (2018). Natural history of brain arteriovenous malformations: systematic review. J Neurosurg Sci..

[10] Shaligram SS, Winkler E, Cooke D, Su H (2019). Risk factors for hemorrhage of brain arteriovenous malformation. CNS Neurosci Ther..

[11] Sforza DM, Kono K, Tateshima S, Vinuela F, Putman C, Cebral JR (2016). Hemodynamics in growing and stable cerebral aneurysms. J Neurointerv Surg..

[12] Shakur SF, Hussein AE, Amin-Hanjani S, Valyi-Nagy T, Charbel FT, Alaraj A (2017). Cerebral arteriovenous malformation flow is associated with venous intimal hyperplasia. Stroke..

[13] Kim H, Al-Shahi Salman R, McCulloch CE, Stapf C, Young WL, Coinvestigators M (2014). Untreated brain arteriovenous malformation: patient-level meta-analysis of hemorrhage predictors. Neurology..

[14] Al-Shahi Salman R, White PM, Counsell CE, du Plessis J, van Beijnum J, Josephson CB (2014). Outcome after conservative management or intervention for unruptured brain arteriovenous malformations. JAMA..

[15] Derdeyn CP, Zipfel GJ, Albuquerque FC, Cooke DL, Feldmann E, Sheehan JP (2017). Management of brain arteriovenous malformations: a scientific statement for healthcare professionals from the American Heart Association/American Stroke Association. Stroke..

[16] Steiger HJ, Fischer I, Rohn B, Turowski B, Etminan N, Hanggi D (2015). Microsurgical resection of Spetzler-Martin grades 1 and 2 unruptured brain arteriovenous malformations results in lower long-term morbidity and loss of quality-adjusted life-years (QALY) than conservative management--results of a single group series. Acta Neurochir (Wien)..

[17] Yang W, Wei Z, Wang JY, Hung AL, Caplan JM, Braileanu M (2016). Long-term outcomes of patients with giant intracranial arteriovenous malformations. Neurosurgery..

[18] Steinberg GK, Do HM, Levy RP, Marks MP, Marcellus ML, Chang SD (2003). Multimodality treatment of giant intracranial arteriovenous malformations. Neurosurgery..

[19] Reinard KA, Pabaney AH, Basheer A, Phillips SB, Kole MK, Malik GM (2015). Surgical management of giant intracranial arteriovenous malformations: a single center experience over 32 years. World Neurosurgery..

[20] Cenzato M, Boccardi E, Beghi E, Vajkoczy P, Szikora I, Motti E (2017). European consensus conference on unruptured brain AVMs treatment (Supported by EANS, ESMINT, EGKS, and SINCH). Acta Neurochir (Wien)..

[21] Ma L, Chen XL, Chen Y, Wu CX, Ma J, Zhao YL (2017). Subsequent haemorrhage in children with untreated brain arteriovenous malformation: higher risk with unbalanced inflow and outflow angioarchitecture. Eur Radiol..

[22] Ma L, Kim H, Chen XL, Wu CX, Ma J, Su H (2017). Morbidity after hemorrhage in children with untreated brain arteriovenous malformation. Cerebrovasc Dis..

[23] Pabaney AH, Reinard KA, Kole MK, Seyfried DM, Malik GM (2016). Management of arteriovenous malformations in the elderly: a single-center case series and analysis of outcomes. J Neurosurg..

[24] Lawton MT, Kim H, McCulloch CE, Mikhak B, Young WL (2010). A supplementary grading scale for selecting patients with brain arteriovenous malformations for surgery. Neurosurgery..

[25] Jiao Y, Lin F, Wu J, Li H, Wang L, Jin Z (2018). A supplementary grading scale combining lesion-to-eloquence distance for predicting surgical outcomes of patients with brain arteriovenous malformations. J Neurosurg..

[26] Han SJ, Englot DJ, Kim H, Lawton MT (2015). Brainstem arteriovenous malformations: anatomical subtypes, assessment of “occlusion in situ” technique, and microsurgical results. J Neurosurg..

[27] Nozaki K, Hashimoto N, Kikuta K, Takagi Y, Kikuchi H. Surgical applications to arteriovenous malformations involving the brainstem. Neurosurgery. 2006;58(4 Suppl 2):ONS-270-8; discussion ONS-8-9. 10.1227/01.NEU.0000210001.75597.81 16582650

[28] Sun Y, Jin H, Li Y, Tian Z (2017). Target embolization of associated aneurysms in ruptured arteriovenous malformations. World Neurosurg..

[29] Pollock BE, Gorman DA, Brown PD (2004). Radiosurgery for arteriovenous malformations of the basal ganglia, thalamus, and brainstem. J Neurosurg..

[30] Martinez JL, Macdonald RL (2015). Surgical strategies for acutely ruptured arteriovenous malformations. Front Neurol Neurosci..

[31] Deng Z, Chen Y, Ma L, Li R, Wang S, Zhang D, et al. Long-term outcomes and prognostic predictors of 111 pediatric hemorrhagic cerebral arteriovenous malformations after microsurgical resection: a single-center experience. Neurosurg Rev. 2020. 10.1007/s10143-019-01210-4 32078085

[32] Potts MB, Lau D, Abla AA, Kim H, Young WL, Lawton MT (2015). Current surgical results with low-grade brain arteriovenous malformations. J Neurosurg..

[33] Bir SC, Maiti TK, Konar S, Nanda A (2016). Overall outcomes following early interventions for intracranial arteriovenous malformations with hematomas. J Clin Neurosci..

[34] Saatci I, Geyik S, Yavuz K, Cekirge HS (2011). Endovascular treatment of brain arteriovenous malformations with prolonged intranidal Onyx injection technique: long-term results in 350 consecutive patients with completed endovascular treatment course. J Neurosurg..

[35] Alexander MD, Hippe DS, Cooke DL, Hallam DK, Hetts SW, Kim H (2018). Targeted embolization of aneurysms associated with brain arteriovenous malformations at high risk for surgical resection: a case-control study. Neurosurgery..

[36] Wang MZ, Qiu HC, Wang S, Cao Y, Zhao M, Zhao JZ (2018). A New Technique for transvenous embolization of brain arteriovenous malformations in hybrid operation. Chin Med J (Engl)..

[37] Tu J, Stoodley MA, Morgan MK, Storer KP. Responses of arteriovenous malformations to radiosurgery: ultrastructural changes. Neurosurgery. 2006;58(4):749-758; discussion -58. 10.1227/01.NEU.0000192360.87083.90 16575339

[38] Cohen-Inbar O, Starke RM, Lee CC, Kano H, Huang P, Kondziolka D (2017). Stereotactic radiosurgery for brainstem arteriovenous malformations: a multicenter study. Neurosurgery..

[39] Mathis JA, Barr JD, Horton JA, Jungreis CA, Lunsford LD, Kondziolka DS (1995). The efficacy of particulate embolization combined with stereotactic radiosurgery for treatment of large arteriovenous malformations of the brain. AJNR Am J Neuroradiol..

[40] Natarajan SK, Ghodke B, Britz GW, Born DE, Sekhar LN (2008). Multimodality treatment of brain arteriovenous malformations with microsurgery after embolization with onyx: single-center experience and technical nuances. Neurosurgery..

[41] Han PP, Ponce FA, Spetzler RF (2003). Intention-to-treat analysis of Spetzler-Martin grades IV and V arteriovenous malformations: natural history and treatment paradigm. J Neurosurg..

[42] Sun DQ, Carson KA, Raza SM, Batra S, Kleinberg LR, Lim M (2011). The radiosurgical treatment of arteriovenous malformations: obliteration, morbidities, and performance status. Int J Radiat Oncol Biol Phys..

[43] Gruter BE, Mendelowitsch I, Diepers M, Remonda L, Fandino J, Marbacher S (2018). Combined endovascular and microsurgical treatment of arteriovenous malformations in the hybrid operating room. World Neurosurg..

[44] Ren Z, Wang S, Xu K, Mokin M, Zhao Y, Cao Y, et al. The working road map in a neurosurgical Hybrid Angio-Surgical suite------ development and practice of a neurosurgical Hybrid Angio-Surgical suite. Chinese Neurosurgical Journal. 2018;4(1). 10.1186/s41016-017-0108-1 PMC7393899 32922868

