# Supplementary material for: Contemporary management of brain arteriovenous malformations in mainland China: a web-based nationwide questionnaire survey
Source: Chin Neurosurg J. 2020 Sep 1;6:26. doi: 10.1186/s41016-020-00206-0 (PMC7461270; doi:10.1186/s41016-020-00206-0)
Supplement: Supplementary file 1 — Additional file 1. Contemporary web-based nationwide questionnaire survey on brain arteriovenous malformations management in mainland China [file 41016_2020_206_MOESM1_ESM.docx]

**Contemporary web-based nationwide questionnaire survey on brain arteriovenous malformations management in mainland China**

Thank you for your participation in this web-based questionnaire survey, which aims to summarize the experiences and treatment progress of brain arteriovenous malformations (bAVMs) in mainland China.

General characteristics

- What’s your name (open question)?
- What’s your institution (open question)?
- Are you the department director of your department (single choice question)?

O Yes

O No

- What’s your professional title (single choice question)?

O Chief physician

O Deputy chief physician

O Attending physician

O Resident physician

- How many bAVMs are treated in your institution every year (open question)?
- What’s your specialty?

O Neurosurgeon

O Neurointerventionist

O Radiosurgeon

General questions

1. Did you agree with the conclusions of ARUBA that conservative management is better than intervention for unruptured bAVMs and could these conclusions be generalized to all unruptured bAVMs (single-choice question)?

O Yes

O No

1. What do you think are the risk factors for subsequent hemorrhage in unruptured bAVMs (multichoice question)?

O Flow-related aneurysms

O Deep venous drainage

O Arteriovenous fistula (AVF)

O Periventricular location

O Small nidus

1. Do you think unruptured Spetzler-Martin (SM) grade IV-V bAVMs are the contraindications of intervention (single-choice question)?

O Yes

O No

If not, which kind of unruptured SM IV-V bAVMs should take interventional treatment (multichoice and open question)?

O Patients with hemorrhagic risk factors

O Patients with epilepsy

O Patients with compact nidus

O Patients with superficial cortical nidus

O Patients with non-eloquent nidus

O Younger patient

Supplement______

1. Do you think giant bAVMs (>6cm) are the contraindication of intervention (Single choice)?

O Yes

O No

If not, which kind of intervention modality do you recommend (multichoice question)?

O Microsurgical resection

O Target embolization (hemorrhagic predictors)

O Curative embolization

O Stereotactic radiosurgery

O Microsurgery + Embolization

O Microsurgery + Radiosurgery

O Embolization + Microsurgery

O Embolization + Radiosurgery

O Radiosurgery + Embolization

O Radiosurgery + Microsurgery

O Combination of the above three single intervention modality

1. Do you think pediatric bAVMs are the contraindication of intervention (single-choice question)?

O Yes

O No

Do you think pediatric intervention strategies should be more aggressive than adults (single-choice question)?

O Yes

O No

And which kind of intervention modality do you recommend for pediatric bAVMs (multichoice question)?

O Microsurgical resection

O Target embolization (hemorrhagic predictors)

O Curative embolization

O Stereotactic radiosurgery

O Microsurgery + Embolization

O Microsurgery + Radiosurgery

O Embolization + Microsurgery

O Embolization + Radiosurgery

O Radiosurgery + Embolization

O Radiosurgery + Microsurgery

O Combination of the above three single intervention modality

Do you agree that the purpose of intervention in pediatric bAVMs is complete nidus obliteration and maximum functional protection (single-choice question)?

O Yes

O No

1. Do you think elderly bAVMs (>65 years) are the contraindication of intervention (single-choice question)?

O Yes

O No

If no, which kind of intervention modality do you recommend (multichoice question)?

O Microsurgical resection

O Target embolization (hemorrhagic predictors)

O Curative embolization

O Stereotactic radiosurgery

O Microsurgery + Embolization

O Microsurgery + Radiosurgery

O Embolization + Microsurgery

O Embolization + Radiosurgery

O Radiosurgery + Embolization

O Radiosurgery + Microsurgery

O Combination of the above three single intervention modality

Do you recognize that partial occlusion and target embolization concentrated on hemorrhagic risk factors were more recommended than complete obliteration for elderly bAVMs (single-choice question)?

O Yes

O No

1. Do you think eloquent AVMs are the contraindication of intervention (single-choice question)?

O Yes

O No

Which intervention modality do you prefer (multichoice and open question)?

O Microsurgical resection

O Target embolization (hemorrhagic predictors)

O Curative embolization

O Stereotactic radiosurgery

O Microsurgery + Embolization

O Microsurgery + Radiosurgery

O Embolization + Microsurgery

O Embolization + Radiosurgery

O Radiosurgery + Embolization

O Radiosurgery + Microsurgery

O Combination of the above three single intervention modality

Supplement______

1. What treatment strategy would you prefer for bAVMs with/without hemorrhagic risk factors located in the internal capsule, basal ganglia, thalamus and brainstem (multichoice and open question)?

bAVMs with hemorrhagic risk factors

O Microsurgical resection

O Target embolization (hemorrhagic predictors)

O Curative embolization

O Stereotactic radiosurgery

O Microsurgery + Embolization

O Microsurgery + Radiosurgery

O Embolization + Microsurgery

O Embolization + Radiosurgery

O Radiosurgery + Embolization

O Radiosurgery + Microsurgery

O Combination of the above three single intervention modality

Supplement______

bAVMs without hemorrhagic risk factors

O Microsurgical resection

O Target embolization (hemorrhagic predictors)

O Curative embolization

O Stereotactic radiosurgery

O Microsurgery + Embolization

O Microsurgery + Radiosurgery

O Embolization + Microsurgery

O Embolization + Radiosurgery

O Radiosurgery + Embolization

O Radiosurgery + Microsurgery

O Combination of the above three single intervention modality

Supplement______

1. When do you think is the best intervention timing in patients with stable ruptured bAVMs (vital signs are stable, no obvious signs of cerebral hernia) (single-choice question)?

O Acute phase (within 48 hours)

O Subacute phase (7 days to 1 month)

O Chronic phase (1 month to 3 months)

O Recovery phase (> 3months)

Subgroup detail questions

If you are neurosurgeon, please went to Question 10 and 11.

If you are neurointerventist, please went to Question 10, 11 and 12.

If you are radiosurgeon, please went to Question 13 and 14.

1. Do you think that the single-stage combined embolization and microsurgery strategy is beneficial (single-choice question)?

O Yes

O No

If yes, which of the following is the most important (single-choice question)?

O Intraoperative angiography (clarify angioarchitecture characteristics and avoid lesion residue) O Intraoperative embolization.

If you select intraoperative embolization, which embolization strategy do you prefer (multichoice and open question)?

O Target embolization (hemorrhagic predictors)

O Embolization of the deep part nidus

O Excessive embolization

Supplement______

1. Which of the following areas do you prefer to embolize in the single-stage combined embolization and microsurgery (single-choice question)?

O The feeding arteries

O The feeding arteries and nidus.

And what’s the reason of your choice (open question)?

______

1. What is the embolic material routinely used for bAVMs in your department (single-choice question)?

O Onyx (eV3, Inc.)

O NBCA or IBCA

O Others

And which embolization strategy do you think is more beneficial (single-choice question)?

O Embolize hemorrhagic risk factors

O Embolize hemorrhagic risk factors and the nidus

1. What is the minimum margin dose you suggested for single-stage radiosurgery (open question)?
2. Do you agree that pre-radiosurgery embolization is not conducive to the subsequent obliteration after radiosurgery (single-choice question)?

O Yes

O No

Which kind of bAVMs do you think would benefit from pre-radiosurgery embolization (multichoice and open question)?

O Patients with hemorrhagic risk factors

O Patients with high flow

O Patients with high-level lesions (SM IV-V)

O Patients with large-volume nidus

Supplement______

Thank you for your cooperation and support for this questionnaire survey.
